# Pyogenic spondylodiscitis of the lumbar spine related to anastomotic fistula after surgery for esophageal cancer: a case report

**DOI:** 10.1186/s40792-020-00922-w

**Published:** 2020-06-30

**Authors:** Yuichi Akama, Takeshi Matsutani, Nobutoshi Hagiwara, Hiroki Umezawa, Tsutomu Nomura, Hidetsugu Hanawa, Keisuke Mishima, Nobuhiko Taniai, Hiroshi Yoshida

**Affiliations:** 1 Department of Gastrointestinal Hepato-Biliary-Pancreatic Surgery, Nippon Medical School, 1-1-5 Sendagi, Bunkyo-ku, Tokyo, 113-8603 Japan; 2 Department of Digestive Surgery, Nippon Medical School Musashi Kosugi Hospital, 1-396 Kosugimachi, Nakahara-ku, Kawasaki-shi, Kanagawa 211-8533 Japan; 3 Department of Plastic, Reconstructive and Aesthetic Surgery, Nippon Medical School, 1-1-5 Sendagi, Bunkyo-ku, Tokyo, 113-8603 Japan

**Keywords:** Pyogenic spondylodiscitis, Esophageal cancer, Cervical anastomotic fistula

## Abstract

**Background:**

Pyogenic spondylodiscitis is an extremely rare complication of esophagectomy for esophageal cancer.

**Case presentation:**

A 70-year-old Japanese man, with a previous medical history of type 2 diabetes mellitus, coronary artery disease, and laryngeal cancer, received neoadjuvant chemotherapy and underwent thoracoscopic esophagectomy with gastric tube reconstruction for advanced esophageal cancer. Cervical esophagogastrostomy with circular-stapled end-to-side anastomosis was performed. However, partial necrosis in the gastric tube developed to form refractory anastomotic fistula. Two months after the initial surgery, debridement and free jejunal transfer reconstruction with the pectoralis major muscle flap were performed. Although the postoperative course of the second surgery was uneventful, the patient complained of severe lower back pain and fever. The patient was diagnosed with pyogenic spondylodiscitis according to the results of the magnetic resonance imaging. *Enterobacter cloacae* were isolated from the arterial blood culture. Sensitive antibiotics were administered continuously, and the patient required to use a lumbar corset for 2 months. Subsequently, his physiological signs and symptoms had completely disappeared.

**Conclusion:**

To the best of our knowledge, this case study is the first study that reported pyogenic spondylodiscitis of the lumbar spine, a complication of cervical anastomotic fistula after surgery for advanced esophageal cancer.

## Introduction

Pyogenic spondylodiscitis is a rare infection of the spine caused by the hematogenous spread of infection or direct inoculation of pathogens from the adjacent purulent focus. The diagnosis of this condition is usually difficult because of the lack of specific findings. The presence of systemic diseases such as diabetes mellitus, liver dysfunction, and renal dysfunction is a risk factor [1–3]. Cases of pyogenic spondylitis of the cervical spine that occurs after treatment for esophageal and laryngeal cancer/trauma have been discussed in previous studies [4–6]. Furthermore, a few reports have revealed that pyogenic spondylitis of the cervical spine is related to anastomotic fistula that occurs after surgery for esophageal cancer [7, 8]. Herein, we report the case of pyogenic spondylodiscitis of the lumbar spine that occurred after surgery for advanced esophageal cancer. The occurrence of such condition, which is a complication of surgery for esophageal cancer, is extremely rare.

## Case report

**Fig. 1:**
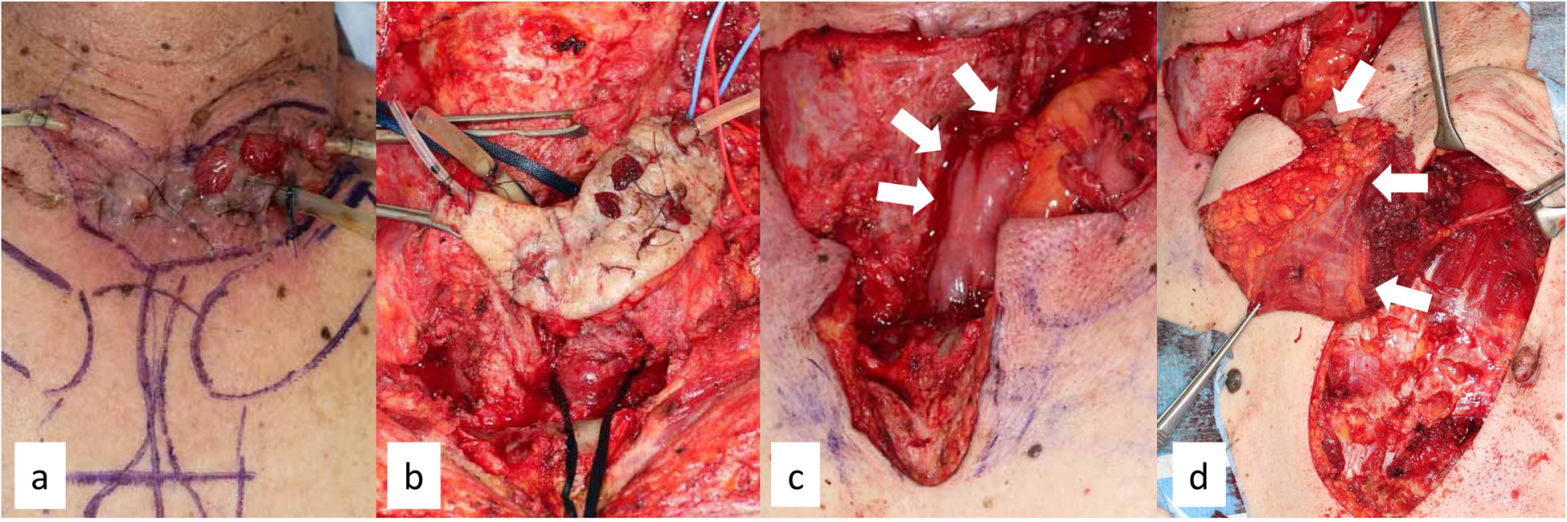
**a** Refractory anastomotic fistula was observed on the 60th day after esophagectomy. **b** Debridement of the necrotized tissues was performed. **c** Salvage reconstruction with the free jejunal autograft (arrows) was performed. The jejunal artery and vein were anastomosed to the right transverse cervical artery and the right internal jugular vein, respectively. **d** The pectoralis major muscle flap (arrows) was used. These figures have been previously published (Umezawa H. Treatment of infections in the head, neck and facial area. PEPARS. 2017; 129:32-9)

**Fig. 2:**
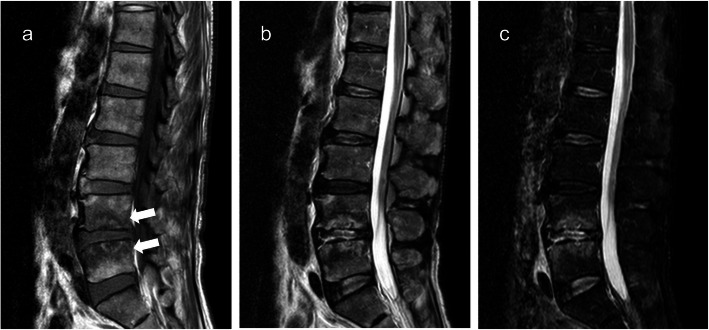
Magnetic resonance images. T1-weighted image (**a**), T2-weighted image (**b**), and STIR image (**c**) revealed pathological changes in the L4 and L5. The signal intensity in the diseased area was low on the T1-weighted image (**a**, arrowheads) and high on the T2-weighted and STIR images (**b**, **c**)

A 70-year-old Japanese man who complained of dysphagia was admitted to our hospital. He had a medical history of using medications for type 2 diabetes mellitus, percutaneous coronary intervention for coronary artery disease, and radiotherapy for laryngeal cancer. Upper gastrointestinal endoscopy revealed an ulcerative and infiltrative type of tumor in the middle of the thoracic esophagus. Chest computed tomography (CT) scan did not reveal the presence of any lymph nodes and distant metastases. The diagnosis was T3N0M0 esophageal squamous cell cancer. The patient received two courses of adjuvant chemotherapy comprising 5-fluorouracil (5-FU) and docetaxel plus cisplatin (DCF regimen; 5-FU 600 mg/m^2^: days 1–5, docetaxel 60 mg/m^2^: day 1, and cisplatin 60 mg/body: day 1). Thoracoscopic esophagectomy, gastric tube reconstruction via the retrosternal route, and cervical esophagogastrostomy with circular-stapled end-to-side anastomosis were performed. On the 4th postoperative day (POD), flare, pus discharge, and saliva outflow from the cervical wound were observed, and cervicotomy with a wide opening was performed. Upper gastrointestinal endoscopy revealed a partial defect in the necrotic wall of the gastric tube at the anastomosis, and air bubbles were appearing in the cervical wound. The patient was initially treated conservatively with drainage tube placement and antibiotic intravenous (i.v.) administration of tazobactam/piperacillin (4.5 g) three times a day, for establishing of a controlled infection around the anastomotic leakage. The patient received nutritional management with a combination of central venous and tube feeding, and blood glucose levels were controlled with insulin administration. Body weight and serum albumin levels were 50 kg and 3.1 g/dL on the 30th POD and recovered to 50.5 kg and 3.4 g/dL after 4 weeks, respectively. At the same time, HbA1c was unchanged from the preoperative level (6.6%) and serum C-reactive protein was close to normal level. However, the patient developed refractory anastomotic fistula, and *Enterobacter cloacae* was isolated from the culture of the cervical wound. On the 60th day following the initial surgery, the left sternoclavicular joint was partially resected to widen the surgical field, and necrotized tissue debridement and free jejunal autograft transfer were performed as salvage reconstruction. The jejunum was transected at the level of the second jejunal vessel, and was joined to the distal end of the cervical esophagus and the proximal end of the residual gastric tube in an end-to-end anastomosis. The jejunal artery and vein were anastomosed to the right transverse cervical artery and the right internal jugular vein, respectively. Further, the free jejunal autograft was covered with the pectoralis major muscle flap (Fig. 1). In addition, a blood-rich pectoralis major muscle flap and two suction drain placements eliminated dead space to prevent osteomyelitis. A feeding jejunostomy tube was placed for nutrition. However, on the 15th day following the second surgery, the patient complained of gradual worsening of lumbago. Upon clinical examination, the patient was febrile a temperature of 38.2 °C, and he was experiencing pain in the lumbar spinal area. Neurological examination revealed weakness in both the legs. However, the sensibility and perception of the patient did not change. The following laboratory examination results were obtained: white blood cell count (WBC) count 9200 /μL (normal range 4000–8500 /μL) and C-reactive protein (CRP) level 9.86 mg/dL (normal range < 0.03 mg/dL). The presence of inflammatory foci was not observed on chest radiography and abdominal ultrasonography. *E. cloacae* was isolated from the arterial blood cultures. Magnetic resonance imaging (MRI) of the lumbar spine at the L4–5 revealed an obvious decrease in the signal intensity on T1-weighted images and an increase in the signal intensity on T2-weighted and short tau inversion recovery (STIR) images, as confirmed based on the typical appearance of pyogenic spondylodiscitis (Fig. 2). The infection might be attributed to the hematogenous spread of the infection from the refractory anastomotic fistula in the neck. After intensive treatment with intravenous antibiotics, tazobactam/piperacillin 4.5 g (i.v.) three times a day for 4 weeks and cefepime 1 g (i.v.) twice a day for the succeeding 4 weeks, oral antibiotic (trimethoprim and sulfamethoxazole 4 g/day for the last 4 weeks) was administered until the levels of the laboratory markers, including WBC and CRP, normalized. Spinal immobilization via lumbar fixation using a corset was continued. The patient’s lumbago and pyrexia gradually diminished, and the CRP and WBC levels decreased. Oral intake was started on the 14th POD, which was the time when the anastomotic fistula closed. The patient took small amounts of food orally. However, he remained dependent on the feeding jejunostomy for most of his nutritional intake. After 12 weeks of antibiotic administration, the patient was free from pain, and no signs of infection were observed. Thus, the administration of antibiotics was discontinued. The patient was discharged from our hospital and was receiving maintenance therapy for cancer. Eventually, he recovered. However, pleural disseminations were observed on the chest CT scan that was conducted during the follow-up 2 years after the initial surgery. The patient died 50 months after the initial surgery. However, the symptoms of infectious and neurological disorders did not recur until the patient’s death.

## Discussion

Patients presented with cervical esophagogastric anastomosis after surgery for esophageal cancer are at the highest risk for ischemia and fistula formation because cervical anastomosis is the farthest from the right gastric and gastroepiploic arteries, which nourish the gastric tube. Refractory anastomotic fistula after surgery is a serious condition, and salvage reconstruction in cervical esophagogastrostomy remains a challenging procedure for gastrointestinal surgeons. Although the patient in the current study had a medical history of diabetes mellitus and coronary artery disease, these conditions are not contraindications to surgery for esophageal cancer. However, patients with these comorbidities may have a higher risk of postoperative morbidity. Moreover, those receiving radiotherapy for laryngeal cancer may have risk factors for cervical tissue damage and impaired wound healing after surgical treatment (simple suturing, resection, and resection with reconstruction). Salvage reconstruction with free jejunal autograft is considered necessary for patients with anastomotic fistula, which cannot be treated with conservative therapy. To prevent the direct spread of pathogens, we used the tissues of the pectoralis major muscle to cover the anterior aspect of the vertebrae. However, in the current case, subsequent spondylitis of the lumbar spine had occurred.

Cases of cervical pyogenic spondylitis occurring after esophageal and laryngeal trauma have been discussed in previous studies. The occurrence of esophageal/hypopharyngeal rupture [9, 10] and iatrogenic esophageal perforation [11] has also been observed. Cervical pyogenic spondylitis and abscess formation after esophageal trauma caused by metal stent placement for benign stricture [12] or unresectable cancer [5, 6] have been observed in previous studies. However, these postoperative sequelae after esophagectomy are extremely rare. Kakuta and Mecklenburg have reported an extremely rare case of esophagospinal fistula with cervical spondylodiscitis after esophagectomy with gastric tube [7, 8]. These patients had received neoadjuvant chemotherapy or chemoradiotherapy for carcinoma of the thoracic esophagus, which is associated with immunosuppression. Kakuta et al. [7] have reported that intraoperative injury in the prevertebral fascia is a causative factor. In 1995, Iannettoni et al. [13] have reported that among 856 patients who underwent a cervical esophagogastric anastomosis after transhiatal esophagectomy, 3 (*n* = 1, vertebral body osteomyelitis; *n* = 2, epidural abscess with neurologic impairment) presented with cervical osteomyelitis. Two of these patients died. Because the most common causes of pyogenic spondylitis include direct spread from esophageal surgery, perforation, or presence of a contiguous focus, all previous cases might involve the cervical spine. However, in general, the causes of pyogenic spondylitis are (1) hematogenous spread of bacteria, (2) direct infiltration by trauma or orthopedic surgery, and (3) continuous infection from nearby soft tissues, of which hematogenous spread is the most common. In our case, no direct damage to the prevertebral fascia was observed during salvage reconstruction surgery. Our patients would be susceptible to infection by immune-compromised state due to diabetes mellitus, the past medical history for radiation therapy, and malnutrition after esophageal surgery. Fortunately, no direct damage to the prevertebral fascia was observed during salvage reconstruction surgery, and cervical pyogenic spondylitis was not developed by direct infiltration of bacteria. *E. colacae* was detected in blood cultures and infected to distant lumbar spines, and we diagnosed pyogenic spondylitis due to hematogenous spread of bacteria.

When neck or back pain and neurological deficit occur in patients with highly advanced esophageal cancer, the differential diagnosis includes metastatic spinal tumor. MRI is the most sensitive and specific modality for the early confirmation and accurate diagnosis of spondylitis [1, 14]. It revealed that spondylitis and metastatic spinal tumors are observed in the areas with decreased signal intensity on T1-weighted images and increased signal intensity on T2-weighted images [1, 14]. Purulent spondylitis often involves the intervertebral disc. However, this structure has normal signal intensity in patients with metastatic spinal tumor.

The principles of treatment include the administration of appropriate antibiotics and recommendation for patients to rest completely during the early stage. *Staphylococcus aureus* is the most commonly isolated pathogen that causes infection after invasive spinal procedures [1, 3, 10, 15]. *E. cloacae* is a member of the normal gut flora in humans and is not usually the pathogen that primarily causes spondylitis. *E. cloacae* infections are usually observed in immunocompromised patients. The use of cefeprime or gentamicin is recommended. Moreover, previous studies have revealed that administration of antibiotics at a duration of < 4–8 weeks is associated with a significantly higher rate of recurrence compared with treatment for > 12 weeks [15–17]. Therefore, the dosing period of antibiotic use is > 12 weeks. Treatment selection is most important in patients with limited life expectancy. The patient’s quality of life might be impaired by prolonged spondylitis and early cancer relapse.

## Conclusion

Purulent spondylitis after esophagectomy is an extremely rare complication that can lead to catastrophic sequelae, such as quadriplegia and subsequent epidural abscess. All esophageal surgeons should be knowledgeable of the disease to facilitate early detection and adequate treatment. When such patients develop recurrent back pain postoperatively, the diagnosis of pyogenic spondylitis must be considered.

## Data Availability

The data are not available for public access due to patient privacy concerns but are available from the corresponding author on reasonable request.

## Abbreviations

CT: Computed tomography
5-FU: 5-Fluorouracil
POD: Postoperative day
i.v.: Intravenous
WBC: White blood cell count
CRP: C-reactive protein
MRI: Magnetic resonance imaging
STIR: Short tau inversion recovery

## References

[1] Cottle L, Riordan T (2008). Infectious spondylodiscitis. J Inf Secur.

[2] Friedman JA, Maher CO, Quast LM, McClelland RL, Ebersold MJ (2002). Spontaneous disc space infections in adults. Surg Neurol.

[3] Osenbach RK, Hitchon PW, Menezes AH (1990). Diagnosis and management of pyogenic vertebral osteomyelitis in adults. Surg Neurol.

[4] Honda K, Asato R, Tsuji J, Kanda T, Watanabe Y, Mori Y (2013). Pyogenic spondylodiscitis after transoral surgery for oropharyngeal cancer. Auris Nasus Larynx.

[5] Lloyd D, Smith D (2002). Cervical discitis in a patient with an oesophageal stent for carcinoma. Rheumatology (Oxford).

[6] Mersol JV, Kozarek RA (2002). Spine complications of stent placement. Gastrointest Endosc.

[7] Kakuta T, Kosugi S, Kanda T, Hatakeyama K (2010). Purulent spondylitis related to anastomotic fistula after esophageal cancer surgery. Interact Cardiovasc Thorac Surg.

[8] Mecklenburg I, Probst A, Messmann H (2008). Esophagospinal fistula with spondylodiscitis and meningitis after esophagectomy with gastric pull-up. Journal of gastrointestinal surgery : official journal of the Society for Surgery of the Alimentary Tract.

[9] Matsuo M, Rikimaru F, Higaki Y, Masuda M (2016). A case of hypopharyngeal cancer with stenosis, perforation, and pyogenic spondylitis development after chemoradiotherapy. Int J Surg Case Rep.

[10] Metcalfe S, Morgan-Hough C (2009). Cervical epidural abscess and vertebral osteomyelitis following non-traumatic oesophageal rupture: a case report and discussion. Eur Spine J.

[11] Mattingly WT, Dillon ML, Todd EP (1982). Cervical osteomyelitis after esophageal perforation. South Med J.

[12] Boulis NM, Armstrong WS, Chandler WF, Orringer MB (1999). Epidural abscess: a delayed complication of esophageal stenting for benign stricture. Ann Thorac Surg.

[13] Iannettoni MD, Whyte RI, Orringer MB (1995). Catastrophic complications of the cervical esophagogastric anastomosis. J Thorac Cardiovasc Surg.

[14] Dagirmanjian A, Schils J, McHenry MC (1999). MR imaging of spinal infections. Magn Reson Imaging Clin N Am.

[15] Grados F, Lescure FX, Senneville E, Flipo RM, Schmit JL, Fardellone P (2007). Suggestions for managing pyogenic (non-tuberculous) discitis in adults. Joint Bone Spine.

[16] Jensen AG, Espersen F, Skinhoj P, Frimodt-Moller N (1998). Bacteremic *Staphylococcus aureus* spondylitis. Arch Intern Med.

[17] McHenry MC, Easley KA, Locker GA (2002). Vertebral osteomyelitis: long-term outcome for 253 patients from 7 Cleveland-area hospitals. Clin Infect Dis.

